# Enhancing nutritional environments through access to fruit and vegetables in schools and homes among children and youth: a systematic review

**DOI:** 10.1186/1756-0500-7-422

**Published:** 2014-07-04

**Authors:** Rebecca Ganann, Donna Fitzpatrick-Lewis, Donna Ciliska, Leslea J Peirson, Rachel L Warren, Paul Fieldhouse, Mario F Delgado-Noguera, Sera Tort, Steven P Hams, Maria José Martinez-Zapata, Luke Wolfenden

**Affiliations:** 1 Effective Public Health Practice Project, McMaster University, Room HSC 3 N25, 1280 Main Street West, Hamilton, Ontario L8S 4 K1, Canada; 2 Manitoba Healthy Living, Youth & Seniors, Manitoba, Canada; 3 Departamento de Pediatria, Facultad Ciencias de la Salud, Universidad del Cauca, Colombia, Popayan, Colombia; 4 Iberoamerican Cochrane Centre, Institute of Biomedical Research (IIB Sant Pau), Barcelona, Spain; 5 Public Health, Gloucestershire Primary Care Trust, Cheltenham, UK; 6 Centro Cochrane Iberoamericano-Servicio de Epidemiologia Clínica, IIB Sant Pau. Pavelló 18. Planta 0 Sant Antoni Ma Claret, 167, 08025 Barcelona, Spain; 7 School of Medicine and Public Health, University of Newcastle, Callaghan, Australia

**Keywords:** Food environment, Fruit and vegetables, Systematic review

## Abstract

**Background:**

Low fruit and vegetable (FV) consumption is one of the top 10 global risk factors for mortality, and is related to increased risk for cancer, cardiovascular disease and diabetes. Many environmental, sociodemographic and personal factors affect FV consumption. The purpose of this review is to examine the effects of interventions delivered in the home, school and other nutritional environments designed to increase FV availability for five to 18-year olds.

**Methods:**

The search included: 19 electronic bibliographic databases; grey literature databases; reference lists of key articles; targeted Internet searching of key organization websites; hand searching of key journals and conference proceedings; and consultation with experts for additional references. Articles were included if: in English, French and Spanish; from high-, middle-, and low-income countries; delivered to anyone who could bring about change in FV environment for 5 to 18 year olds; with randomized and non-randomized study designs that provided before-after comparisons, with or without a control group. Primary outcomes of interest were measures of FV availability.

**Results:**

The search strategy retrieved nearly 23,000 citations and resulted in 23 unique studies. Interventions were primarily policy interventions at the regional or state level, a number of curriculum type interventions in schools and community groups and a garden intervention. The majority of studies were done in high-income countries.

The diversity of interventions, populations, outcomes and outcome measurements precluded meta-analysis. The most promising strategies for improving the FV environment for children are through local school food service policies. Access to FV was successfully improved in four of the six studies that evaluated school-based policies, with the other two studies finding no effect. Broader state or federally mandated policies or educational programs for food service providers and decision makers had mixed or small impact. Similarly family interventions had no or small impact on home accessibility, with smaller impact on consumption.

**Conclusions:**

The studies have high risk of bias but more rigorous studies are difficult to impossible to conduct in naturalistic settings and in policy implementation and evaluation. However, there are promising strategies to improve the FV environment, particularly through school food service policies.

## Background

According to the World Health Organization (WHO) low fruit and vegetable (FV) consumption is one of the top 10 global risk factors for mortality [1]. In 2010 inadequate FV consumption accounted for 4.9 million (fruit) and 1.8 million (vegetables) deaths globally [2]. Increased FV consumption plays a significant protective role in the prevention of cancer and chronic diseases, such as cardiovascular disease and diabetes, and is also positively related to overall health status. Dietary patterns rich in FV (i.e., providing anywhere from 5 to 13 servings of FV each day depending on caloric requirements) significantly decrease disease risk and burden [3]. The WHO estimates that 2.7 million lives could be saved annually by increasing individual FV consumption to the recommended 400 g per day. Such an increase in consumption would also decrease the worldwide non-communicable disease burden by 1.8% [4].

A recent systematic review of determinants of FV consumption among children and adolescents, identified availability and accessibility of FV in the home was positively associated with increased consumption after controlling for individual socio-demographic factors [5]. Reviews have also identified a number of community-level environmental factors that may impede access to FV which have been associated with intake including physical, economic and social factors; country wide supply, availability and accessibility; availability of FV in stores in the local community, schools and community-based programs; and multi-level policies for increasing access to FV [5-7]. Prior to implementing this systematic review, the authors conducted a scoping review to identify and map literature that has evaluated the effects of community-based interventions designed to increase FV access and/or consumption among 5 to 18 year olds [8]. With many reviews already available about consumption [9,10] and obesity prevention [11,12], we identified a gap in the literature related to interventions to improve the food environment, particularly at school and at home. Many people, regardless of country of origin and income status, do not meet recommended guidelines for FV intake. However, consumption behavior is a result of the interplay of multiple variables at the individual level (e.g., sociodemographic, psychosocial and perceived nutrition environment) and environmental level (e.g., community nutrition environment, organizational nutrition environment, and consumer nutrition environment), both of which are also influenced by policies (global, national, or local) and the information environment [13]. Both individual and ecological variables must be considered in the design of interventions to improve the FV environment as part of initiative to enhance child FV intake. Enhanced understanding of relevant intervention research, implementation and impact on both FV access and chronic disease health indicators will provide guidance to public health decision-makers and policy-makers in the establishment and maintenance of effective policies and programs to support children’s nutritional status.

The determinants of FV consumption are many and complex [13]. Interventions that influence the upstream determinants of consumption have the potential to move beyond an individual level focus to impact population level food environments and food consumption patterns [14]. Using Glanz and colleagues' framework [13], the purpose of this review is to examine the effects of interventions in the organizational nutrition environments (at home, school and other) designed to increase FV availability of five to 18-year olds.

## Methods

### Search

We searched the following 19 databases up to June 2012:

MEDLINE and Pre-MEDLINE (from 1966); EMBASE (from 1980); CINAHL and Pre-CINAHL (from 1982); the Cochrane Central Register of Controlled Trials (CENTRAL); the Cochrane Public Health Group Specialized Register; PsycINFO (from 1967); Dissertation Abstracts (from 1980); ERIC (from 1966); Effective Public Health Practice Project Database (1998); Sociological Abstracts (1952); Applied Social Sciences Index (1987); CSA Worldwide Political Science Abstracts (1975); ProQuest (ABI/Inform Global) (1923); PAHO Institutional Memory Database (1902); WHO Database on Child Growth and Malnutrition; Healthstar; Current Contents; ScienceDirect; and LILACS. Search terms were adapted according to the requirements of individual databases in terms of subject heading terminology and syntax.

The search strategy for MEDLINE is shown in the Additional file 1.

The World Health Organization database and the Global Health Database were searched for relevant grey literature. Reference lists of all relevant articles were hand searched for additional relevant references. In our contact with authors of included studies, we asked for a list of other potentially relevant articles. These lists were reviewed for additional relevant references.

We conducted a targeted Internet search of key organization websites, including the World Health Organization ( http://www.who.int/en/), the Food and Agriculture Organization of the United Nations ( http://www.fao.org/), and Pan American Health Organization ( http://new.paho.org/).

We hand-searched the following 15 journals (for the 12-month time period prior to the initial electronic database search in August 2010) based on our consultation with experts in the field to determine rich publication sources: Health Policy; Journal of Public Health Policy; Journal of Health Politics, Policy, and Law; Health Economics, Policy, and Law; American Journal of Clinical Nutrition; Journal of Health Services Research; American Journal of Public Health; Journal of the American Dietetic Association; Nutrition Reviews; Maternal and Child Nutrition; Nutrition and Dietetics; Nutrition Research; Public Health Nutrition; American Journal of Preventive Medicine; and Journal of Human Hunger.

To identify additional relevant references we consulted with policy-makers and researchers with experience in promoting, implementing and studying strategies to improve the FV environment for children.

### Inclusion/exclusion criteria

We sought articles in any language. Articles in English, French, and Spanish were reviewed for inclusion, assessment, and data extraction for inclusion in the review; however, we did not have capacity to translate articles in other languages for inclusion in the review.

Randomized and non-randomized controlled trials (including cluster-controlled trials, controlled time series), studies with interrupted time series designs (to assess changes that occur over time), and before-after studies with comparison groups (including those with historical controls) were included in the review. The study had to report both baseline and outcome data. The clusters within studies that answer this review question include school units, classrooms and communities rather than individuals as the unit of analysis.

This review included populations from low-, middle-, and high-income countries and focused on children aged 5 to 18 years since childhood is a critical time period for establishing food habits and routines. We included interventions delivered to anyone or any institution that can bring about change in FV environment for 5 to18 year olds (i.e., parents, communities and others within the population, including the children/adolescents themselves). This age group was chosen because another review on this topic, focusing on children under the age of five, was underway and has recently been published [15].

Interventions included those aimed at modifying the FV environment through provision of FV, policies, and/or education: child nutrition programs such as breakfast/lunch and summer food service programs; community programs (e.g., community gardens); economic supplements and subsidies to purchase FV, including subsidies for schools and food stamp programs; environmental school change strategies (e.g., changing the types of foods provided in cafeterias or vending machines, nutrition-friendly school initiatives); environmental interventions/industry partnerships focused on point-of-purchase (e.g., restaurants, grocery store distributors and retailers); campaigns to draw attention to healthier products in grocery stores or to highlight the health benefits of certain foods; Internet, telephone and media interventions; farm-to-school programs that use locally produced foods; social marketing campaigns; policies that affect accessibility factors (e.g., agricultural policies), or seek to increase FV consumption (i.e., school board level, provincial/national level).

Acceptable settings included: homes, schools, health department settings, religious institutions, family/child centres, community/recreation centres, non-governmental organizations, and primary healthcare settings. We excluded programs or strategies delivered through hospitals; outpatient clinics located within hospital settings; commercial programs, such as Health Check; universities/colleges; and metabolic or weight loss clinics.

Primary outcomes included: FV supply (i.e., market inventory); change in food environment (e.g., at home, at school); FV disappearance/food transition (cafeteria and grocery store sales). Measures could be at the individual, family, school, or community level. Secondary outcomes included consumption of FV; awareness of importance/impact of FV consumption among targeted individuals; attitudes towards consumption of FV; general health measures (including changes in weight); and any reported adverse outcomes or unintended consequences.

### Selection of studies

The search strategy identified titles and abstracts, which were independently examined by two reviewers for relevance. All articles selected by either team member were retrieved for full text review. Two reviewers independently examined the full text of retrieved articles for relevance. A third review author was consulted to resolve disagreements related to inclusion of articles. Two review authors independently assessed risk of bias of each article. Differences were resolved through discussion. Reasons for exclusion were documented and are available from the authors.

### Data extraction

Data were extracted from all included articles on: study design; participant, setting and intervention characteristics; and outcomes.

One reviewer extracted the data and a second verified the data extraction form. A third reviewer resolved discrepancies through discussion. Reviewers attempted to contact lead authors a minimum of three times to obtain missing data. The review authors were not blinded to the names of authors or institutions.

Relevant studies were evaluated for risk of bias using the Cochrane Collaboration’s tool for assessing risk of bias [16]. Two reviewers independently rated articles based on the six criteria: sequence generation, allocation concealment, blinding of participants and personnel, blinding of outcome assessment, attrition and outcome reporting. Each study was rated as ‘low’ , ‘unclear’ or ‘high’ risk of bias, according to the Collaboration’s tool [16]. We were guided by the recommendations in Chapter 13 of the Cochrane Handbook for Systematic Reviews of Interventions for analysis of non-randomized controlled trials [16]. In assessing for ‘other bias’ , reviewers evaluated validity and reliability of data collection tools; appropriateness of statistical analyses and use of intention-to-treat analyses; and whether intervention integrity was described or measured. We used a third review author to resolve disagreements related to assessment of risk of bias.

We attempted to contact 14 authors regarding missing data; we were unable to obtain current contact information for two, two did not respond, and one responded but did not provide the clarifying information requested. We report all statistically significant and non-significant outcomes, however, we were not able to conduct a meta-analysis due to diverse definitions and measurements used.

## Results

**Figure 1:**
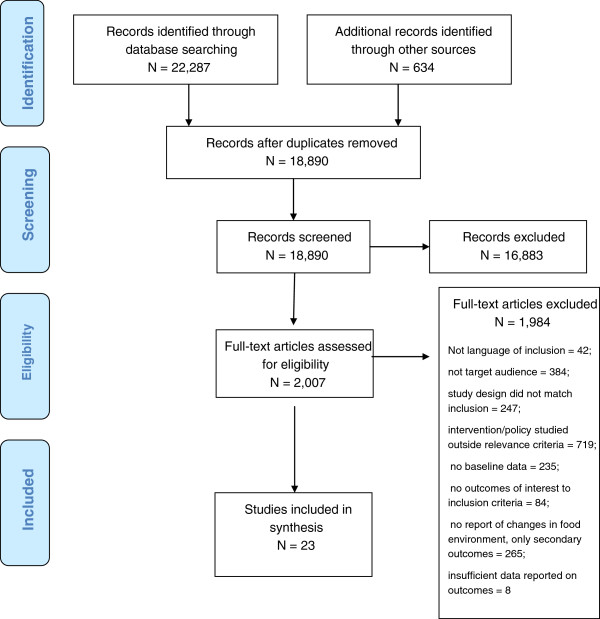
Flow diagram.

The search strategy retrieved nearly 23,000 citations; following full text review, 1,984 (98.8%) studies were excluded with 23 (1.2%) unique studies remaining. See Figure 1 for a flowchart of literature retrieved, levels of screening, included studies and reasons for exclusion at full text screening. See Table 1 for citations found with each search strategy, and Additional file 2 for the Characteristics of Included Studies.

**Table 1:** Sources of citations

**Database searched**	**Database total**	**Percentage of database**
EMBASE	4,698	21.1%
MEDLINE	4,464	20.0%
HealthSTAR	3,544	15.9%
CINAHL	1,674	7.5%
Web of Science	1,533	6.9%
CCTR (Cochrane)	1,337	6.0%
ERIC	799	3.6%
Science Direct	785	3.5%
Social Science	785	3.5%
PsychInfo	751	3.4%
Social Abs	655	2.9%
Dissertation Abstracts	614	2.8%
ASSIA	368	1.7%
ABI/INFORM	151	0.68%
Thesis Dissertations	85	0.38%
Worldwide Political Abstracts	44	0.20%
**Total from electronic databases**	**22,287**	**100%**
Other sources	634	
**Total from all sources**	22,921	

### Study design and intervention location

This review includes one trial that was conducted in American schools [17] and three quasi-experimental studies, one conducted in the United States and the others in South Africa and the Netherlands [18-20]. Five cluster-controlled studies were included, of which three were conducted in the United Kingdom and the other two conducted in the United States. These studies were conducted primarily in schools, with some including home-based components [21-24]; there was one exception wherein the study was conducted within Boy Scout troop and Internet settings [25]. Fourteen before-after studies with no control group were included; 12 conducted in the United States [26-37], one conducted in France [38], and one in England [39].

### Intervention type

Making distinctions to classify intervention type is difficult given the multiple components of some studies. Some interventions that were primarily focused on policy targeted food services and afterschool programs [17,27] while others examined implementation of district level local wellness policies as part of the National School Lunch Program [26,28,37] in terms of their impact on cafeterias, snack bars, vending machines and school-level policies. Two studies examined state-wide approaches including a public school nutrition policy [31], and a program to reduce chronic disease through a multi-faceted approach that included in-school environmental and policy changes associated with nutrition [35]. One study examined the impact of food pricing strategies on cafeteria sales [32] and another multifaceted changes to elementary school food service delivery [33].

Interventions that were more program or curriculum focused included one aimed at teachers [38], food service workers [29], school tuck shop provision [22], curriculum or multifaceted school interventions [23,24,40], after school and family involvement [19,21], web-based curriculum [30], and other community educational programs at locations including Boy Scouts [25], YWCA garden [34]; and YWCA food service [36], and multisite communities [20].

### Outcomes related to food environment

Most included studies reported on changes in the food environment [17,19-21,23-27,29,30,33,34,36-40], with a few exceptions. One study reported on changes to the FV supply [35], while four reported on food sales (e.g., in cafeterias or grocery stores) [22,28,31,32], and three reported on both changes in the food environment and food sales [24,29,33].

### Secondary outcomes reported

In addition to reporting food environment outcomes, 15 studies reported on secondary outcome measures. Of these, most included a measure of consumption [19-23,25,27,28,31,34,35,39,40], with several reporting on knowledge or awareness of the importance of or impact of consumption [20,25,38,40], some reporting on attitudes toward consumption (including self-efficacy) [19,33-35,40] and two reporting general health measures [25,35].

### Target audience(s)

Most studies included children as a target audience, with three exceptions. Two studies targeted the general population [20,35] and one targeted solely school teachers [38]. Some studies targeted both children and their parents [19,21,30,40].

### Risk of bias in included studies

**Figure 2:**
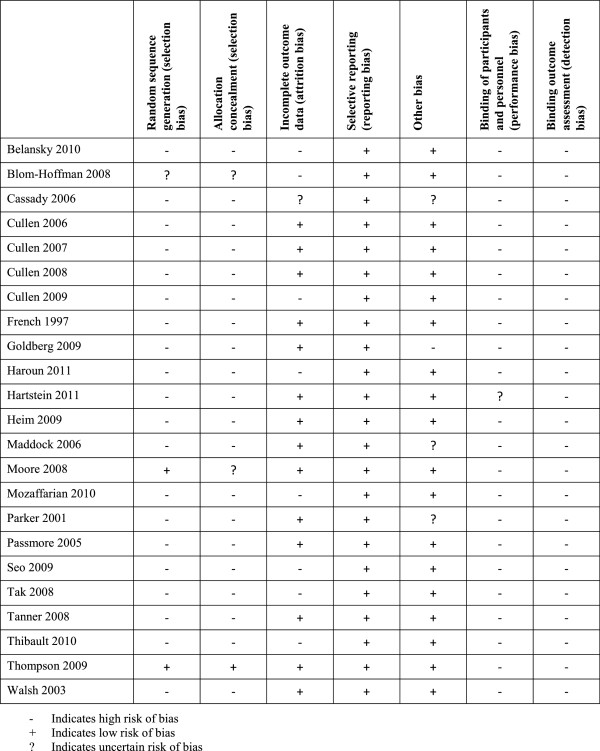
Risk of bias.

Each of the included studies (n = 23) was rated as having high risk of bias (see Figure 2). As such, there is a high risk of bias across studies that impacts confidence in the findings.

### Effects of interventions

The reviewed studies employed a wide variety of programs and policies with either process or outcome goals of improving the fruit or vegetable environment, or both. Outcome measures were unique to each study, with no two studies measuring the exact same outcomes with the same measurement tool. For example, although a number of studies used measures of home FV availability and accessibility, the instruments to measure these outcomes used different scales. Results to measure impacts of program or policy implementation were also highly variable. Some studies found statistically significant improvements in FV availability, accessibility, or both, while several other studies found no significant impacts and one study found inexplicably significant negative changes to school food production of vegetable and salad offerings following the intervention.

### Policy interventions targeting school food service

In a trial involving 30 middle schools, dieticians worked with half the schools to change in-school vending machine offerings to attempt to eliminate 100% fruit juice/sugar-added beverages and decrease dessert offerings with the ultimate goals of increasing fruit consumption at meals and reducing excess fruit juice consumption [17]. The intervention schools successfully eliminated the number of vending machine slots allocated to fruit juice from 31% to 0%, compared to 13% and 42% at baseline and follow-up respectively for the control group.

Implementation of school-based policies was also found effective in impacting food service production and offerings [27,39]. Haroun and colleagues used an uncontrolled before-after evaluation study to examine the impact of Food-Based and Nutrient-Based standards implemented in 136 primary schools in England on lunchtime in-school catering services’ food and drink provision [39]. Overall schools’ catering services increased the percentage of fruit (13% to 16%) and ‘vegetables and salad’ (19% to 23%) provided between baseline and follow-up. In terms of foods selected by children having a school lunch, 14.7% more children took ‘vegetables and salad’ while 6.5% more took fruit and 8.4% more took fruit-based desserts, which contained on average 40% fruit (all statistically significant changes). Following food service changes, more students chose vegetables, fruit, fruit juice and water (P < 0.01); however, observational data showed 1/3 to 2/5 of the portions were wasted.

Using a before-after study Cassady and colleagues evaluated the impact of a school-based policy in California measuring changes in average daily FV servings offered within an after-school program offered at 44 elementary schools following the implementation of an organizational policy [27]. No changes were found in vegetable servings offered in the previous menu as compared to the new menu; however, significant increases were observed in fruit offerings (0.6 servings before to 1.1 average daily servings after, P < 0.05). An unintended consequence was that milk provision decreased post-intervention.

Goldberg et al. also used a before-after study to examine changes in school food services in elementary schools in Somerville, Massachusetts, USA with the goal of obesity prevention among school children [33]. The food service intervention was multi-faceted, involving changes in school meals, professional development and capacity building among food service staff, and communication strategies in partnership with principals, teachers, and media outlets to encourage healthy eating among students. Limited data were reported, however, authors indicate that fresh fruit availability in school meals (breakfast and lunch) increased from twice per week at baseline to five times per week following the intervention.

Cullen et al. [31], using a before-after study, examined the impact of the Texas Public School Nutrition Policy, through examining cafeteria food production data within 47 schools. There were no significant differences in FV served in the cafeteria in daily fruit servings or "regular non-fried vegetables" comparing before and after the policy was implemented. However, significant decreases in high-fat vegetables served were observed post-implementation with primary schools reducing mean daily servings from 0.49 to 0.36 and secondary schools reducing mean daily servings from 0.80 to 0.54. Greater reductions occurred in schools located in larger districts versus smaller districts.

Similarly, Cullen and colleagues used a before-after design to examine the impact of policy changes on weekly school-based snack food sales in three middle schools in Houston, USA, finding no significant changes in FV sales [28]. Daily mean intake of fruit and juices did not change but there was a small, statistically significant reduction in the daily mean servings of vegetables after the intervention was introduced (0.3 to 0.2 mean servings per day, P < 0.05).

### Policy interventions targeting the price of FV

A before-after study examined the impact of food pricing strategies on high school cafeteria sales of FV in two large high schools in Minnesota, USA [32], where prices for fruit, baby carrot, and salad were reduced by 50%. Following the intervention, prices were returned to baseline levels and again measured. Fruit sales significantly increased between the baseline mean of 14.4 pieces sold per week and the low price time period mean of 63.3 pieces sold per week. Sales then significantly decreased to a mean of 26.1 pieces per week when prices reverted to baseline levels. Baby carrot sales were also significantly impacted as a result of the pricing strategy with mean sales initially increasing to 77.6 packets per week from the baseline of 35.6 packets per week, and decreasing to 42.0 packets per week when the pricing strategy reverted. No significant differences in mean servings of salad sold per week were found across the three time periods with the three pricing strategies.

### Policy interventions targeting school and broader food environments

Maddock and colleagues evaluated the impact of a statewide Healthy Hawaii Initiative, legislature focused on the prevention of chronic diseases, using a before-after study design [35]. A multi-faceted program targeting the general public (n = 4,476) was implemented based on the policy change; this program included school-based interventions, a community-based intervention, a public education campaign, and professional education targeting health providers. Resultant changes in the community’s food environment were measured by examining public perceptions of FV affordability, whether FV were easy to buy close to respondents’ homes, and whether local restaurants offered a wide range of FV. Significant improvements in FV affordability were observed between baseline (3.73) and first follow-up at one year (3.84), as well as baseline and second follow-up at two years (3.84). There were no significant changes in close, easy access for purchasing FV across time points; however, significant improvements in restaurant offerings of FV were found between baseline (3.83) and second follow-up (3.93). Although the authors report on these three aspects of perceived environmental variables for nutrition, there is no further description of these measures nor associated scales. The proportion of high school students who consumed at least five servings of fruits and vegetables per day decreased by 4.8% but the proportion of adults who consumed at least five servings increased by 5.25% over the follow-up period (no confidence intervals or P values reported).

In contrast, some studies that examined the impact of policy implementation found no significant changes to the FV environment for children. Belansky et al. used a before-after design to evaluate the impact of the implementation of the federally mandated Local Wellness Policy in 45 rural, low-income elementary schools in Colorado, USA [26]. Improvements were found in fresh fruit but not fresh vegetable lunch choices in school lunchrooms between baseline (2005 to 2006) and follow-up (2007 to 2008).

Seo also evaluated implementation of a Local Wellness Policy in 226 high schools in Indiana, USA [37]. Using a before-after design, he studied changes in secondary school food policies and food preparation practices associated with the policy driven change. No significant changes were found in the percentage of schools with school food policies that offered fruit; lettuce, vegetable or bean salads; or 100% fruit or vegetable juices, as reported by Principals or food service directors for the 226 secondary schools.

### Programs targeting school food service

Cullen and colleagues evaluated a 6-week pilot before-after study testing the feasibility of implementing environmental changes in school food service programs in six middle schools in three American states (North Carolina, Texas and California) [29]. The intervention involved food service changes to achieve 13 goals, several of which related to serving more FV. School food production and sales records were evaluated at baseline and then daily during the pilot study. As process indicators, following the intervention the number of schools offering ‘at least 3 FV menu items/day’ increased from two to six and ‘at least 10 different FV items over each three week period’ increased from one to six in their National School Lunch Program (NSLP) Food. The outcomes of ‘at least one fruit offered per day’ , ‘at least one vegetable offered daily’ , and ‘at least seven different FV items over each three week period’ all increased from zero to six schools. Across the schools, total NSLP FV served increased from 1.10 servings/student/day at baseline to 1.42 servings/student/day. Despite this overall increase across schools, slight decreases were seen in the two California schools and in one school in Texas. No tests of statistical significance were reported.

In a cluster-controlled study, in-school fruit tuck shops were implemented in intervention schools (n = 23) with no tuck shops available in control schools (n = 20) [22]. Due to challenges associated with maintaining accurate records of tuck shop sales, limited sales outcome data were reported.

To examine the effects of an intervention to improve snack offerings based on the Programme national nutrition sante, Thibault and colleagues used a before-after design to study snacks offered by preschool teachers (n = 343 participating schools) in the Aquitaine region of France in two separate surveys (2004 to 2005 and 2007 to 2008) [38]. Differences between surveys found that the offering of snacks had decreased from 68.7% to 57.9% of the teachers, with a decrease from 60% to 40% of teachers offering mostly sweet snacks (P <0.001) and increase from 8.5% to 17.7% offering fruit and/or milk (P <0.001). More teachers offered fruit as a snack, increasing from 2.6% of the teachers to 9.4% (P < 0.001).

Other program evaluation studies found no significant impacts on FV accessibility as a result of intervention implementation.

A cluster-controlled study examined a multifaceted whole school intervention through School Nutrition Action Groups with a goal of improving food provision and choices for adolescents in 12 intervention schools compared to adolescents in 12 control schools who did not receive these groups [24]. Changes in the food environment were examined through school dining room food sales; however, no significant changes in baseline to follow-up (3 months following the 2-year intervention) for potato, vegetable and salad sales were found across intervention and control schools, despite significant increases in main meals and snack meals sold in intervention schools compared to controls. Intervention school students observed more choice in foods available than control students, but no statistical analysis was done on this outcome [24].

In the Peterborough Schools Nutrition Project, a 2-year multifaceted school based program, was implemented and evaluated using a cluster-controlled design [23]. The intervention was implemented in two large schools and included school food groups that were formed to create environmental changes in school-based food provision and foster linkages between nutrition-related curriculum activities and school catering services. The control school, in contrast, received no intervention but was observed over the same 2-year period. Changes in the accessibility of fresh fruit (portions/week) and vegetables and salad (portion/week) produced by school caterers were measured in the two intervention schools and the control school before and after the intervention. The authors acknowledge limitations associated with baseline fruit portions that make results unreliable. However, they found vegetable and salad portions produced per week decreased in both intervention schools and increased in the control school.

### Programs targeting the home and families

A number of studies demonstrated the effectiveness of programs in improving FV access among children 5 to 18 years. Tanner et al. conducted a pilot quasi-experimental study involving an after-school media and nutrition literacy intervention, family fun nights and a media campaign developed by the children in the intervention group and delivered to their parents [19]. This pilot intervention was implemented in elementary schools, targeting upper middle-school students and their parents with the goal of positively impacting in-home nutritional environments and FV. Significant pre- to post-intervention improvements in availability were found in the intervention group versus the control group, with no differences in children’s consumption, self-efficacy or motivation.

Several studies that examined the effects of home food environment interventions failed to demonstrate positive significant changes. A pilot quasi-experimental study examined the long-term effects of the Dutch Schoolgruiten Project to promote FV consumption among primary school children [18]. As part of this study, children in the 31 intervention schools were provided with free fruit or ready-to-eat vegetables semi-weekly together with a school nutrition program that sought to increasing knowledge and skills related to FV consumption compared to control group schools (conditions not described). As part of this intervention study, changes in home food environments were assessed through pre- and post-evaluations of FV availability and children’s ability to take fruit without asking at home. These outcomes were measured through both child-reported and parent-reported questionnaires. At post-intervention, the intervention group had an increased percentage of children reporting FV were usually available at home (71.6% pre and 80.8% post). In the control group (n = 24 schools) there was no significant change in reported FV availability at home. After adjusting for differences between groups at baseline, there was no significant intervention effect on FV accessibility. In the parent reported data, FV availability at home did not change from baseline to follow-up in either group. At a two-year follow-up, there was an intervention effect of increased fruit intake (0.15 servings per day, 95% CI 0.004 to 0.286) but not vegetable intake. Findings from this study were also reported in [41,42].

In 2008 Cullen and colleagues conducted a pilot before-after study that examined the feasibility of an 8-week web-based intervention to promote healthy eating behaviours in 67 families of African American girls aged 9 to 12 based on a modified version of the Texas Expanded Fruit and Nutrition Education Program [30]. The Family Eats intervention was designed for weekly web access to support parents in making positive changes to the home food environment and to promote healthy food choices (i.e., FV). Parent- and child-reported fruit, vegetable and juice availability were used to evaluate changes to the food environment. No significant changes in parent- or child-reported availability of juice, fruit or vegetables were found between pre- and post-intervention surveys.

Blom-Hoffman et al. evaluated a literacy-based, interactive component of a multi-year school-based education program to communicate nutrition information with families using a cluster-controlled design (n = 4 schools) [21]. The control group did not receive the home component of the school-based education program. The intervention sought to increase FV consumption, and also measured parent-reported changes in the food environment at home through the FV Availability/Accessibility scale. There were no significant differences in either FV availability or FV accessibility in the home or servings of FV children ate each day, at one year and two years post-intervention, despite improvement in knowledge scores in the intervention parents compared to the controls (P < 0.05).

### Programs targeting communities or community programs

A large quasi-experimental study including more than 600 participants evaluated the impact of a nutrition education program delivered to one urban and three rural communities and implemented by local nutrition advisors to improve nutrition knowledge and behaviours [20]. Randomly selected households completed structured and previously piloted surveys before and after the intervention. As part of the survey, households were asked to indicate whether they grow their own vegetables (a measure of change in the food environment) and vegetable availability at home. Among the intervention communities, large before-after increases in households reporting growing their own vegetables were found in a large rural area and a small rural area (each greater than 40%; statistically significant before/after change within community), with more modest increases (10% and 11%) found in the urban intervention group and a small rural control group community respectively. Data were not reported for the fourth intervention and second control group communities, both small rural areas.

Another community-based program was evaluated using a cluster-controlled design. The 9 week multi-component 5-a-day Achievement Badge Program intervention implemented in 42 Boy Scout troops to increase FV consumption was comprised of weekly 30-minute in-troop Boy Scout education sessions augmented by 25 minutes of weekly online activities that targeted behaviour change and goal setting [25]. The effectiveness of this intervention was compared to a control group that received a mirror-image intervention focused on increasing physical activity. Fruit juice and vegetable home availability were measured using summary scores from an availability scale before and after the intervention. Both intervention and control groups demonstrated increases in home fruit/fruit juice and vegetable availability from baseline; however, the intervention group had significantly greater mean changes in fruit/fruit juice availability post-intervention than the control (an increase of 1.87 vs. 0.58 items). Both groups increased vegetable availability pre- to post-intervention by one item with a mean score of nine vegetable items available in the home. At immediate post-intervention assessment, there were statistically significant intervention effects for fruit and juice consumption (mean difference of 0.4 servings per day increase in the intervention group over the control group, P = 0.03). These differences disappeared at the six-month follow-up. Findings from this study were also reported in Baranowski 2002 [43] and Baranowski 2006 [44].

Mozaffarian and colleagues evaluated the impact of an organizational change on the quality of snacks and beverages served in 11 Young Men’s Christian Association (YMCA) after-school programs in seven American states using a before-after study design [36]. The organizational change included the implementation of Environmental Standards for Healthy Eating to guide after-school program menus. Menu reports were completed by YMCA staff and included information on food types (e.g., whether FV were fresh, canned, dried, or frozen) and food groups served. Measurements of baseline mean snack and beverage servings per week were 1.9 combined FV, 1.2 fruit, 0.7 vegetables, 1.3 fresh fruit/vegetables, and 0.6 dried, canned or frozen FV. After the intervention the first four measures of mean weekly servings increased significantly to 5.2, 3.2, 1.9, and 3.9 servings respectively; however, no significant changes in weekly servings of dried, canned or frozen FV were found (1.3 servings per week post-intervention). The percentages of caloric contributions to total daily snack and beverage calories by total FV and fresh FV also significantly increased from 7.6% to 22.7% and 4.7% to 15.6% respectively.

A pilot before-after study examined the short-term impact of an educational intervention delivered within a YMCA summer camp to promote FV intake among fourth to sixth grade children (n = 93) [34]. As part of this study, a process evaluation was conducted that examined the short-term impact of the program on home FV availability as reported by the children. Availability was measured using a 7-item scale that assessed frequency of FV availability (mean scores ranged from 1–4) with lower scores indicating less frequently availability. No significant changes in student reported home food availability were found between baseline and follow-up surveys (2 weeks after the intervention) with mean scores of 3.11 and 3.12, respectively. Mean FV availability/accessibility scores as rated by parents, however, increased slightly from 3.1 to 3.2 (rated on a 1–4 scale, P = 0.05). More specifically, baseline vegetable availability in previous two weeks was 5.3 (range: 0–11) and fruit availability in the previous two weeks was 2.9 (range: 0–5) as reported by parents. Significantly increases were found with a mean vegetable availability score of 6.3 (P < 0.001) and fruit availability score of 3.3 (P < 0.05) at follow-up. For children there were statistically significant improvements in total number of FV ever eaten, vegetable preferences, and FV asking behaviour. Findings from this study were also reported in Heim 2011 [45].

## Discussion

There are promising results for specific school food service policies, (vending machine, cafeteria, snack and after school food offerings) with four of six studies finding improved FV environments. In attempting to account for differences in impact, six of seven policy studies included large numbers of schools with only one finding no significant impact on FV [31], and then one study had only three schools and showed no differences [28]. The latter study resulted in a reduction in offering of vegetable servings after the intervention. It may be useful to further explore sample size in future studies. One organizational policy resulted in significant increases in fruit offerings but unexpectedly resulted in decreased milk provision [27]. The reports of unintended results offer no insights or possible explanations for these results.

Inconsistent findings were frequently found according to types of food associated with interventions, often with changes resulting in improved sales or consumption of fruits but not vegetables. For example, fifty per cent price reductions in high school cafeterias for fruit, carrots, and salad increased consumption of fruit and carrots, with a corresponding decrease in consumption when prices reverted to usual. No differences were found across price points for salad [32].

Broader policy interventions aimed at changing decisions of school principals or food service managers had little impact, with one broader community intervention that increased FV affordability and FV offerings in restaurants but decreased consumption among high school students [35]. There are no consistent findings for programs targeting food service with significant improvements in offerings but inconsistency across intervention schools [29]; and decreases in FV availability in intervention schools and increases in control schools [23].

Few programs altered home FV availability. When consumption was assessed, the change was small even when statistically significant, and it would be difficult to determine if the small change would impact other health outcomes. All five studies that assessed knowledge or awareness reported significant improvements. Five of the seven studies reporting change in attitudes found positive results. No studies reported on general health measures (e.g., weight, BMI or serum measures) or on adverse effects of the interventions, except for one that found a decrease in FV consumption [29] and provision of milk [27].

This review provides a narrative synthesis of available international evidence on the effectiveness of interventions to improve the home and school and other FV environments for children aged 5 to 18 years of age. This literature is heavily based on studies conducted within high-income countries, which limits applicability to low- to middle-income contexts. Other than the small number of studies that focused on a particular ethnic target population or comparisons between urban and rural settings, the included studies did not report subgroup differences by gender, ethnicity, or socioeconomic gradients. This limits our understanding of how interventions that were found to be effective could be operationalized in different populations and contexts to address health inequities, and whether similar effects would be found.

For the narrative analysis, the review has classified interventions according to policy or program interventions. However, these interventions take place on a continuum and are often not either/or. The theoretical basis of the interventions was often unstated, with curriculum/program interventions most often stated or inferred to have social learning or behavioural basis. There are insufficient studies that test similar intervention across by ethnicity, socioeconomic gradients or countries, to allow any analysis of different interventions according to these variables.

The overall quality of evidence within the included studies is classified as weak, as every study was determined to have high risk of bias associated with its methodological approach. However, the nature of policy implementation and community-based interventions are such that randomization is rarely feasible, nor is blinding of participants and outcome assessors. The level of rigour in methodology may be close to the highest that could be expected for these sorts of naturalistic studies.

There was large variation in duration of the intervention (one month to three years) and length of follow-up (immediate post-intervention to four year follow-up) with the longer duration and follow up related to policy changes. Many different approaches were taken to assess the FV environment, and within one outcome (like fruit access) the measurement varied across appearance (for example in vending machines), sales, requests, choice and consideration of wastage. This variability of outcomes and measurement did not allow for meta-analysis. Thus the review is limited by the narrative nature of the analysis. Outcome measurements usually were based on measurement tools that had not been tested for reliability and validity. Similarly, self-reported intake was sometimes measured by valid and reliable tools such as 4-day recall. However, they were often based on self-report using questions of unknown reliability and validity. Use of consistent, valid and reliable outcome measures would be a great step forward for research in this area.

Some population-based intervention studies that may have the potential to impact FV accessibility for children were excluded since they did not report outcome measures for children aged 5 to 18 years specifically. It should also be noted that many of the included studies were focused primarily on impacting FV consumption and may not have had FV access as a primary outcome measure, but rather a process indicator or secondary outcome.

## Conclusions

With many reviews available about childhood obesity prevention and treatment, and nutrition more generally, this review adds to knowledge about the state of research about interventions to alter the food environment in schools and homes. The most promising strategies are local school food service policies. The FV environment was successfully improved in four of the six studies that evaluated school-based policies, with the other two studies finding no effect. Broader state or federally mandated policies or educational programs for food service providers and decision makers had mixed or small impact. Similarly family interventions had no or small impact on home accessibility.

Controlled study designs to examine the effects of implementing policies and programs to increase FV accessibility to address health inequities within and across communities, especially those implemented in low- and middle-income contexts would be of particular interest to the field. Controlled study designs are, however, difficult to implement within a naturalistic setting, such as a school, and when evaluating policies. FV policies and programs emerge and evolve in response to contextual factors and, as such, are often not developed as research studies. At the same time, fuller description of contexts within reports and explicit identification of a theoretical basis would be useful. Where randomization is done, authors need to report on sequence generation and allocation concealment. A core of standard food environment or access, and even consumption measurement tools that are reliable and valid would move the field forward.

## Competing interests

No known potential competing interests.

## Authors’ contributions

All authors (RG, DFL, DC, LP, RW, PF, MFD-W, ST, SPH, MJM-Z, LW) contributed their skills and expertise to this review including the protocol development, reviewing included studies, and reviewing manuscripts. All authors read and approved the final manuscript.

## Supplementary Material

Additional file 1 Search strategy. Click here for file

Additional file 2 Characteristics of Included Studies. Click here for file
