# Supplementary material for: Enhancing nutritional environments through access to fruit and vegetables in schools and homes among children and youth: a systematic review
Source: BMC Res Notes. 2014 Jul 4;7:422. doi: 10.1186/1756-0500-7-422 (PMC4114435; doi:10.1186/1756-0500-7-422)
Supplement: Additional file 2 — Characteristics of Included Studies. [file 1756-0500-7-422-S2.docx]

**Additional file 2: Characteristics of Included Studies**

**Belansky 2010**

| **Methods** | Before-after study |
| --- | --- |
| **Participants** | 45 low-income, rural Colorado elementary schools (mean enrolment 204 students) |
| **Interventions** | US government issued mandate under the Child Nutrition and Women, Infants, and Children Reauthorization Act of 2004 requiring school districts participating in the National School Lunch Program to create a Local Wellness Policy (LWP) by June 2006 with intent of increasing opportunities for healthy eating and physical activity. |
| **Outcomes** | Trends in school-level nutrition features in rural, low-income Colorado elementary schools, self-reported by lunchroom and principals via the School Environment and Policy Survey  **Number of daily fresh fruit lunch choices in the lunchroom**  2005-2006: n = 30; Mean +/- standard deviation 0.80+/-0.71  2006-2007: n = 39; Mean +/- standard deviation 0.95+/-0.76  2007-2008: n = 34; Mean +/- standard deviation 1.15+/-0.89  P value 2005 vs. 2007: 0.04 (model includes all observations, e.g., 2005 n = 30 and 2007 n = 34 for fresh fruit choices, but does not include year 2006 observations)  **Number of daily fresh vegetable lunch choices in the lunchroom**  2005-2006: n = 30; Mean +/- standard deviation 1.27+/-0.98  2006-2007: n = 39; Mean +/- standard deviation 1.13+/-0.98  2007-2008: n = 34; Mean +/- standard deviation 1.29+/-1.03  P value 2005 vs. 2007: 0.9 |

**Blom-Hoffman 2008**

| **Methods** | Cluster-controlled |
| --- | --- |
| **Participants** | Intervention: n = 149; Control: n = 148  Four elementary schools in a large, urban, public school district. |
| **Interventions** | The intervention group received the Fruit and Vegetable Promotion Program (FVPP), a school-based, multi-year, multi-component program that also used an interactive, home-based approach to family-school involvement to increase children's FV consumption. The family component involved a literacy-based approach with shared book reading; books were designed to provide multiple opportunities for children to respond through active engagement. Children in this group also participated in Athletes in Service program, a physical activity program in which all schools were already participating.  The control group received only the Athletes in Service intervention. |
| **Outcomes** | FV home availability M (SD) - Parents were asked if they had 48 types of fruits or vegetables in the home in the past 7 days  **Pretreatment**  Intervention: 17.51 (3.85); Control: 17.76 (5.22)  **Post-Year 1**  Intervention: 18.78 (4.04); Control: 19.67 (5.33)  **Post-Year 2**  Intervention: 19.00 (3.84); Control: 18.90 (5.26)  Group x Time Interaction: ns  FV accessibility M (SD) - Parents used the following scale to respond to seven FV accessibility items on a modified version of the Fruit and Vegetable Availability/Accessibility Scale: 1= Never; 2=Once in a While; 3=Most of the Time; 4= All of the Time  **Pretreatment**  Intervention: 2.65 (.58); Control: 2.58 (.63)  **Post-Year 1:**  Intervention: 2.81 (.46); Control: 2.60 (.48)  **Post-Year 2**  Intervention: 2.76 (.56); Control: 2.54 (.53)  Group x Time Interaction: ns |

**Cassady 2006**

| **Methods** | Before-after |
| --- | --- |
| **Participants** | 44 public elementary schools; approximately 8,000 children take part in after-school program |
| **Interventions** | In 2001, Students Today Achieving Results for Tomorrow (START) administrators adopted the Children's 5 a Day-Power Play! curriculum, which teaches children to eat at least 5 daily servings of FV. The following year, they changed their snack vendor and implemented a policy that increased the servings of FV on after school program snack menus to be more consistent with the 5 a day guidelines. |
| **Outcomes** | Fruit servings: Previous menu:0.6; Current menu: 1.1; Change, %: +83  Vegetable servings: Previous menu: 0.0; Current menu: 0.0; Change, %: 0 |

**Cullen 2006**

| **Methods** | Before-after |
| --- | --- |
| **Participants** | 3 middle schools in Houston Texas (n = 2,970) |
| **Interventions** | Food Service Director Implemented local school food policy changes including removal of snack chips, candy, sweet desserts and sweetened beverages from all of the district middle-school snack bars and removal of vending machines from the cafeterias for the 2002-03 school year. |
| **Outcomes** | Results from time series analyses of point-of-service sales: autoregressive integrated moving average (ARIMA) model specification and corresponding model coefficient, standard error (SE) and coefficient test statistic  **Fruit juice and vegetables**  ARIMA model specification: ARIMA (0,1,1)  Model coefficient (SE): -0.12 (3.33); Statistics: t=-0.04, p=0.970  **Percentage of total weekly sales mean (SD)**  Year 1 (pre-implementation) 11.27 (7.91)  Year 2 (during implementation): 1.42 (3.42)  **Servings of fruit and juice mean servings per day (SD)**  Year 1 (pre-implementation) 0.32 (0.25)  Year 2 (during implementation)0.36 (0.3) (not statistically significant)  **Servings of vegetables mean servings per day (SD)**  Year 1 (pre-implementation) 0.3 (0.12)  Year 2 (during implementation) 0.2 (0.09) (p<0.05). |

**Cullen 2007**

| **Methods** | Before-after |
| --- | --- |
| **Participants** | Six middle schools in three states |
| **Interventions** | Intervention with 13 goals targeted all school food environments; five goals related to serving more variety of fresh FV and lower fat entrees in the National School Lunch Program (NSLP) and a la carte lines.  Intervention 1 (North Carolina): NSLP menus followed the traditional food-based menu planning system, with the offer-versus-serve option whereby a student must take at least three to five offered components for the meal to qualify as a reimbursable meal.  Intervention 2 (Texas): the menus followed the NuMenu nutrient standard meal planning system, which utilized computer software to ensure the meals met specific nutrient standards. The menu consisted of at least two entrees, three side dishes (e.g., fruit, vegetables) and milk.  Intervention 3 (California). School 1 used the food-based menu planning system and offer-versus-serve. A daily salad bar was counted as a reimbursable meal item. There was one express NSLP line, three a la carte lines, and a chip/snack cart outside. A 4-oz fruit juice was available as a reimbursable meal item daily. School 2 used the NuMenu system and offer-versus-serve. A 4-oz fruit juice was available as a reimbursable meal item daily. |
| **Outcomes** | National School Lunch Program FV served per student per day during the baseline week and during week six  **California**  School 1: baseline 2.93; at 6 weeks 2.67  School 2: baseline 0.83; at 6 weeks 0.82  **North Carolina**  School 1: baseline 0.50; at 6 weeks 0.81  School 2: baseline 0.26; at 6 weeks 0.82  **Texas**  School 1: baseline 0.25; at 6 weeks 1.90  School 2: baseline 1.60; at 6 weeks 1.51  Overall change: baseline 1.10; at 6 weeks 1.42  Also reports for fruits, vegetables and juice separately. |

**Cullen 2008**

| **Methods** | Before-after |
| --- | --- |
| **Participants** | 67 families (a parent and a 9-12 year old daughter) |
| **Interventions** | Family Eats, web-based nutrition intervention for African American families designed to be accessed once a week by parents to improve the home food environment and promote healthy food choices, particularly FV. Parents viewed specified activities, delivered in a planned sequence each week. |
| **Outcomes** | FV availability in the home was measured for 12 frequently consumed fruits, 3 juices, and 18 vegetables.  **Mean (SD) for Family Eats! Scales**  Availability of juice: Baseline 1.39 (0.30); Post 1.37 (0.31)  Availability of fruit: Baseline 1.56 (0.21); Post 1.49 (0.24)  Availability of vegetables: Baseline 1.42 (0.20); Post 1.40 (0.18)  **Child Scales**  Availability of juice: Baseline 1.49 (0.33); Post 1.38 (0.32)  Availability of fruit: Baseline 1.58 (0.23); Post 1.55 (0.24)  Availability of vegetables: Baseline 1.50 (0.23); Post 1.47 (0.23) |

**Cullen 2009**

| **Methods** | Before-after |
| --- | --- |
| **Participants** | 47 schools:  19 primary schools; 28 secondary schools  23 schools in small districts; 24 schools in large districts |
| **Interventions** | Texas Public School Nutrition Policy, implemented statewide in fall 2004 restricts the portion sizes of high-fat and sugar snacks to fewer than 200 kilocalories per serving package and sweetened beverages to 12 ounces or less, limits the fat content of milk offered to 1% or less, provides guidelines for the fat content of foods served, and sets limits on the frequency of serving high-fat vegetables such as french fries |
| **Outcomes** | No significant improvement in fruit servings between school years (pre and post implementation)  **Average for all schools: Mean (SD)**  Fruit (2003-2004): 0.54 (0.32)  (2004-2005): 0.57 (0.35)  Regular vegetables (2003-2004): 0.64 (0.32)  (2004-2005): 0.63 (0.35)  Outcomes also reported separately for primary schools, secondary schools, small districts and large districts |

**French 1997**

| **Methods** | Before-after |
| --- | --- |
| **Participants** | Two high schools: School 1 (n = 1,431); School 2 (n = 1,935) |
| **Interventions** | The intervention consisted of a pricing strategy to affect sales of FV in an adolescent population. Fruit, carrot and salad purchases were monitored in two high school cafeterias for an initial baseline period; prices were then reduced by 50% and sales were monitored. Finally, prices were returned to baseline and sales were monitored for an additional three weeks. |
| **Outcomes** | **Mean (+/- standard error) number of items sold per week by experimental period**  Fruit (pieces): Baseline 1: 14.4 +/- 14.0; Low Price: 63.3 +/- 14.2; Baseline 2: 26.1+/- 14.2; p=0.0005  Carrots (packets): Baseline 1: 35.6 +/- 49.0; Low Price: 77.6 +/- 49.2; Baseline 2: 42.0 +/-49.2; p=0.01  Salads (servings): Baseline 1: 14.6 +/- 9.5; Low Price: 16.0 +/-9.6; Baseline 2: 16.0 +/- 9.6; p=0.85 |

**Goldberg 2009**

| **Methods** | Before-after |
| --- | --- |
| **Participants** | Elementary school food service staff, students, parents, teachers, and school leaders |
| **Interventions** | School food service intervention consisting of school meal changes (menu adjustments including increased quantity and variety of produce at breakfast and lunch), professional development and capacity building and communication strategies. |
| **Outcomes** | Menu changes in Somerville Public Schools Results from the Shape Up Somerville Intervention, 2003-2004 School Year  Fresh fruits and breakfast (frequency per week): Pre-intervention: 2; Intervention: 5  Fresh fruits at lunch (frequency per week): Pre-intervention: 2; Intervention: 5  Entree salads (grades 4-8) at lunch (frequency per week): Pre-intervention: 2; Intervention: 3  Side salads (grades 1-3) at lunch (frequency per week): Pre-intervention: <1; Intervention: 1 |

**Haroun 2011**

| **Methods** | Before-after study |
| --- | --- |
| **Participants** | n = 139 primary and "middle-deemed" schools at baseline; n = 136 schools at follow up  All schools 100+ pupils |
| **Interventions** | Survey of nationally representative sample of primary schools by School Food Trust in England in order to determine impact of new food-based standards (FBS) in terms of catering provision of food and drink at lunchtime, pupils' choices and consumption of food and drink at lunchtime, the nutrient content of school lunches and compliance of provision with the standards for school food; compared with results of similar survey executed from before implementation (2005). |
| **Outcomes** | Compared with 2005, schools in 2009 were providing significantly more vegetables and salad (4.0%; p<0.001), fruit (3.0%; p<0.001) and fruit juice (1.2%; p<0.001) |

**Hartstein 2011**

| **Methods** | Randomized controlled trial |
| --- | --- |
| **Participants** | 30 middle schools: 15 middle schools in each group with 11 schools/group at follow-up. |
| **Interventions** | Research dietitians worked with school nutrition programs at each intervention school to identify acceptable vending items that met the two study goals. Implemented over a two year period.  The nutrition component goals specific to vending machines were to: serve only dessert and snack foods with = 200 kcals per single-serving package; and to eliminate 100% fruit juice and beverages with added sugar, with the exception of flavored nonfat or 1% milk in volumes of 12 fluid ounces or less. The availability of 100% fruit juice was restricted in vending machines to encourage consumption of fruit at meals and to reduce excess fruit juice consumption |
| **Outcomes** | Changes in food environment: number of vending machine slots allocated to each food/beverage group category (beverages with added sugar, water, milk, 100% fruit juice, artificially sweetened beverages and snack foods) was summed and the percentage of total slots for each category was calculated |
| **Notes** | This work was completed with funding from NIDDK/NIH grant numbers U01-DK61230, U01-DK61249, U01-DK61231, and U01-DK61223 to the STOPP-T2D collaborative group, ClinicalTrials.gov number, NCT00458029. |

**Heim 2009**

| **Methods** | Before-after study |
| --- | --- |
| **Participants** | n = 93; 93% completed both baseline and post-assessment |
| **Interventions** | The Delicious and Nutritious Garden: Pilot intervention for a 12 week YMCA summer camp for 4th to 6th grade children aiming to promote FV intake. Children participated in garden-based activities twice a week and educational activities such as taste test, preparation of FV snack and family newsletters occurred weekly. |
| **Outcomes** | Child survey assessed availability using a 7-item scale with response categories of hardly ever, sometimes, often, and almost always:  **Influence of the Delicious and Nutritious Garden on home availability: (rating range 1-4) mean+/- standard deviation**  Baseline: 3.11+/-0.54  Follow-up: 3.12+/-0.57  p=0.721  **Total number of fruits ever eaten (SD)**  Baseline: 4.72 (0.61)  Follow-up:4.86 (0.41)  p= 0.02  **Total number of vegetables ever eaten (SD)**  Baseline: 7.8 (2.24)  Follow-up: 9.17 (2.09)  p<0.001  **Vegetable preferences (SD)**  Baseline: 3.17 (0.75)  Follow-up: 4.3 (0.71)  p< 0.001  **Fruit and vegetable asking behaviour (SD)**  Baseline: 2.16 (0.47)  Follow-up: 2.32 (0.51)  p=0.02  **Parental valuing and FV consumption**  Baseline: 3.5 (0.5)  Follow-up: 3.7 (0.5)  p= 0.05 |

**Maddock 2006**

| **Methods** | Before-after study |
| --- | --- |
| **Participants** | General public: 4,495 participants at first follow-up; 4,476 participants at final follow-up. |
| **Interventions** | Four interventions: community-based, school based, public education and professional education.  School-based intervention: grants to create systems, environmental and policy changes for tobacco use, nutrition, and physical activity using the eight component model devised by the CDC for SCHPs. This program included training school personnel through annual leadership institutes and hands-on technical assistance.  Community-based interventions: planning grants to improve nutrition, increase physical activity, and reduce and prevent tobacco use.  Public Education Campaign: launched in 2002 with television and radio advertisements, advertisements in movie theatres, press releases, a website and special events with the message that small achievable changes in eating and getting active can add up to enormous health benefits directly to communities statewide.  Professional Education: PiTCH program worked with health care providers and other health-related groups to review national guidelines and programs for smoking cessation, nutrition and exercise counselling. |
| **Outcomes** | three questions about perceived food environment - FV are (1) affordable (2) easy to buy close to where I live (3) wide range of FV served in local restaurants. Perceived environment was a measure of the individual’s belief about the surrounding environment.  **Affordability (SD)**  Baseline: 3.73 (1.4)  Follow-up 1 year: 3.84 (1.35)  Follow-up 2 years: 3.84 (1.4)  p<0.001  **Fruits and vegetables are easy to buy close to where I live**  Baseline: 4.58 (0.94)  Follow-up 1 year: 4.61 (0.91)  Follow-up 2 years: 4.59 (0.92)  not significant  **Restaurants in my area offer a wide range of fruits and vegetables**  Baseline: 3.83 (1.37)  Follow-up 1 year: 3.89 (1.41)  Follow-up 2 years: 3.93 (1.37)  p<0.01  **Per cent adults eating five or more servings FV per day**  Baseline: 22.4%  Follow-up 1 year: 27.6%  (SD and P values not given)  **Per cent teens eating five or more servings FV per day**  Baseline: 20.1%  Follow-up 1 year: 15.3%  (SD and P values not given)  **Subjective norms**  Baseline: 5.4 (3.1)  Follow-up 1 year: 6.52 (3.3)  Follow-up 2 years: 6.19 (3.15)  p<0.001  **Self-efficacy**  Baseline: 7.51 (2.86)  Follow-up 1 year: 7.52 (3.0)  Follow-up 2 years: 7.41 (2.95)  not significant |
| **Notes** | This project was funded by the Hawaii Tobacco Settlement Special Fund through a contract with the Hawaii Department of Health |

**Moore 2008**

| **Methods** | Cluster-controlled |
| --- | --- |
| **Participants** | 23 intervention schools (n = 918 students); 20 control schools (n = 714 students)  Schools were primary and junior schools in eight local education authorities in south-west England and south Wales. |
| **Interventions** | Intervention schools operated fruit tuck shops throughout one academic year; control schools did not. |
| **Outcomes** | A detailed analysis of sales data was not undertaken.  Over the 23 schools, there were 5,600 students on the schools rolls, which over an academic year totals approximately 1.1 million student school days. Fruit sales over the year in the 23 intervention schools were estimated to have been in the region of 70,000 fruits. This is the equivalent of 0.06 fruits per student per day, which in turn equates to approximately 1 in 4 children eating one piece of fruit per week, or 1 in 17 eating fruit every day.  Consumption was self-reported:  **Portions of fruit consumed at school**  Intervention: 0.74; Control: 0.69; not significant  **Portions of fruit consumed in a 24 hour period.**  Intervention: 2.54; Control: 2.51; not significant  **Portions of sweets, chocolate and biscuits consumed at school**  Intervention: 1.12; Control: 1.01; not significant  **Portions of sweets, chocolate and biscuits consumed in a 24 hour period.**  Intervention: 3.95; Control: 3.81; not significant |

**Mozaffarian 2010**

| **Methods** | Before-after |
| --- | --- |
| **Participants** | 11 original YMCA sites |
| **Interventions** | YLC (YMCA Learning Collaborative) was a multifaceted organizational change initiative with an after-school component aiming to integrate health promotion and enhanced communication practices into existing programs; program was evaluated according to implementation of specified healthy eating standards: 1) offering fresh fruit or vegetable options daily, 2) not serving foods with trans fats, 3) offering water as a primary beverage daily 4) not serving sugar sweetened beverages 5) serving more whole grains instead of processed grains. Also offer 100% juice and low-fat or skim milk two or three times each per week. |
| **Outcomes** | Average baseline and post-intervention snack and beverage servings per week: 7 YMCA Learning Collaborative (YLC) sites, United States, 2005-2006  **Number of servings/week - Mean (SD)**  FV - Baseline: 1.9 (2.2); Post-implementation: 5.2 (1.8); Mean change 3.3; Post YLC vs. Baseline .009  Fruits - Baseline: 1.2 (1.6); Post-implementation: 3.2 (1.4); Mean change: 2.0; Post-YLC vs. Baseline: .02  Vegetables - Baseline: 0.7 (0.6); Post-implementation: 1.9 (1.3); Mean change: 1.2; Post-YLC vs. Baseline: .048  Fresh fruits/vegetables - Baseline:1.3 (1.8); Post-implementation: 3.9 (1.8); Mean change:2.6; Post-YLC vs. Baseline: .02  Dried, canned, frozen - Baseline: 0.6 (0.6); Post-implementation: 1.3 (0.8); Mean change: 0.7; Post-YLC vs. Baseline: .14 |

**Parker 2001**

| **Methods** | Cluster-controlled |
| --- | --- |
| **Participants** | School 1 (Intervention) n = 1,029  School 3 (Intervention) n = 1,077  School 2 (Control) n = 1,091 |
| **Interventions** | Intervention Groups:  School Food Groups (SFG), school-based groups consisting of staff, caterers and health professionals, providing a forum for initiating positive changes in the food provision and eating environment within the school, and also to facilitate better links between nutrition education and food provision were established. The areas targeted were the establishment of communication networks, pupil involvement, food availability and eating environment.  Catering - food availability: strategies to increase the availability and uptake of healthier food choices using discreet techniques to promote these choices. The areas targeted were proportion and position of healthier options  Catering - eating environment: positive changes to make the eating environment more consumer friendly and attractive in order to encourage the pupils to eat in school. The areas targeted were provision of fast food areas for healthier options, provision of crockery, shorter queues and entertainment  Peer-related and curriculum activities: Areas targeted were use of drama in workshops and/or as a production and input to planned Personal and Social Education (PSE) lessons on food and health. |
| **Outcomes** | Number of portions per week or proportions of food groups produced by school caterers and the 95% CIs  **Fresh fruit (portions/week)**  School 1: Baseline: 153*; Post-intervention: 60; (95% CI 4 to 154)  School 3: Baseline: 132*; Post-intervention: 'very little'; (95% CI -35 to 255)  School 2: Baseline: 33; Post-intervention: 32; (95% CI 83 to 247)  **Vegetables and salad (portions/week)**  School 1: Baseline: 281; Post-intervention: 132; (95% CI 50 to 283)  School 3: Baseline: 150**; Post-intervention: 50**; (95% CI -9 to 239)  School 2: Baseline: 120; Post-intervention: 230; (95% CI 83 to 247)  *Baseline figures for fruit were very different from the subsequent figures provided by caterers throughout the study, so that interpretation of these figures is unreliable  **At School 3, figures were provided for vegetables only and do not include salad. |

**Passmore 2005**

| **Methods** | Cluster-controlled |
| --- | --- |
| **Participants** | Intervention schools (n = 12); Control schools (n = 12)  All schools were urban |
| **Interventions** | Using results of baseline pupil questionnaire, School Nutrition Action Groups (comprised of school senior management, teacher(s), school cook and possibly an Area Catering Manager and 8-10 pupils) were expected to direct and implement changes to the food provision in the school, communicate with school about the changes being made; enable additional work to support the changes to the food provision to occur and administer a follow-up survey to investigate whether pupils could identify changes to the food and drink provision. |
| **Outcomes** | Food sales in schools at baseline and follow-up  **Potatoes, vegetables, salad - Mean (SD)**  Intervention: Baseline 198.9 (118.4); Follow-up 184.6 (107.6)  Control: Baseline 68.2 (40.6); Follow-up 65.9 (44.6)  ANOVA between groups: Not significant |

**Seo 2009**

| **Methods** | Before-after |
| --- | --- |
| **Participants** | 226 schools (198 public high schools and 28 non-public high schools) |
| **Interventions** | Congress passed Section 204 of Public Law 108-265, the Child Nutrition and Re-authorization Act of 2004. This legislation required each local education agency (LEA) or school district participating in the National School Lunch Program or School Breakfast Program to develop a local wellness policy by July 1, 2006. |
| **Outcomes** | Changes in school food policy in Indiana high schools (n = 150)  Offers fruit: Pre-implementation: 113 (75%); Post-implementation: 114 (76%)  Offers lettuce, vegetable or bean salads: Pre-implementation: 106 (71%); Post-implementation: 112 (75%)  Offers 100% fruit juice or 100% vegetable juice: Pre-implementation: 124 (83%); Post-implementation: 126 (84%) |

**Tak 2008**

| **Methods** | Quasi-experimental |
| --- | --- |
| **Participants** | 31 intervention schools; 24 control schools |
| **Interventions** | Children in the intervention group received a piece of fruit or ready-to eat vegetables for free twice a week at the mid-morning break.  Additionally, a school curriculum, developed and pre-tested by the Netherlands Nutrition Center Foundation that aimed to increase knowledge and skills related to FV consumption, was offered to intervention schools. The intervention schools were not obliged to use this curriculum, but they were encouraged to do so. |
| **Outcomes** | Determinants of fruit intake in the intervention and control groups, at baseline and at second follow-up, conducted on both parent- and child-reported data: Schoolfruiten Project, a Dutch primary school-based intervention providing free fruit and vegetables  **Child-reported data**  **Is the child allowed to take fruit without asking**  No: Intervention Group: Baseline (n = 27, 8.3%); Follow-up 2: (n = 6; 1.8%); p<0.001  Control Group: Baseline (n = 48, 12.2%); Follow-up 2: (n = 17, 4.3%); p<0.001  Sometimes: Intervention Group: Baseline (n = 72, 22.1%); Follow-up 2: (n = 43; 13.2%)  Control Group: Baseline (n = 134; 34.2%); Follow-up 2: (n = 78; 19.7%)  Yes: Intervention Group: (n = 227, 69.6%); Follow-up 2: (n = 277; 85%)  Control Group: (n = 210; 53.6%); Follow-up 2: (n = 301; 76.0%)  **Fruit available at home**  Never/Sometimes: Intervention Group: Baseline (n = 93; 28.4%); Follow-up 2: (n = 63; 19.2%); p=0.001  Control Group: Baseline (n = 80; 20.3%); Follow-up 2: (n = 61; 15.3%); p=0.037  Usually: Intervention Group: Baseline (n = 235, 71.6%); Follow-up 2: (n = 265; 80.8%)  Control Group: Baseline (n = 315; 79.7%); Follow-up 2: (n = 227; 84.7%)  **Parent-reported data**  **Is the child allowed to take fruit without asking**  No: Intervention Group: Baseline (n = 12;8.3%); Follow-up 2: (n = 8; 5.5%); p=0.026  Control Group: Baseline (n = 27, 9.6%); Follow-up 2: (n = 7; 2.5%); p<0.001  Sometimes: Intervention Group: Baseline (n = 25; 17.2%); Follow-up 2: (n = 15; 10.3%)  Control Group: Baseline (n = 55; 19.6%); Follow-up 2: (n = 38; 13.4%)  Yes: Intervention Group: (n = 108; 74.5%); Follow-up 2: (n = 122; 84.1%)  Control Group: (n = 198; 70.7%); Follow-up 2: (n = 238; 84.1%)  **Fruit available at home**  Never/Sometimes: Intervention Group: Baseline (n = 9; 6.3%); Follow-up 2: (n = 5; 3.5%); p=0.157  Control Group: Baseline (n = 9; 3.2%); Follow-up 2: (n = 15; 5.3%); p=0.059  Usually: Intervention Group: Baseline (n = 135; 93.8%); Follow-up 2: (n = 139; 96.5%)  Control Group: Baseline (n = 272; 96.8%); Follow-up 2: (n = 269; 94.7%)  **Child report on fruit intake (pieces per day)**  Intervention Group: Baseline 1.74 (1.12): Follow-up 2 1.52 (0.91)  Control Group: Baseline 1.73 (1.13) ; follow-up 2 1.34 (0.87)  p <0.005  **Parent report on fruit intake (pieces per day)**  Intervention Group: Baseline 1.2 (0.81): Follow-up 2 1.37 (0.86)  Control Group: Baseline 1.09 (0.77); follow-up 2 1.14 (0.82)  p=0.008  **Child report of vegetable intake (grams/day)**  Intervention Group: Baseline 113.5 (60.3): Follow-up 2 1.02 (46.3)  Control Group: Baseline 103.5 (50.4); follow-up 2 95 (41.1)  p=0.025  **Parent report of vegetable intake (grams/day)**  Intervention Group: Baseline 74.8 (34.5): Follow-up 2 83.6 (39.1)  Control Group: Baseline 65.1 (29.6); follow-up 2 75.8 (35)  p=0.050 |

**Tanner 2009**

| **Methods** | Pilot, Quasi-experimental |
| --- | --- |
| **Participants** | 18 intervention students and 21 control students |
| **Interventions** | Intervention group participated in the 6-week after-school program consisting of 12 2-hour sessions including two interaction nutrition sessions, two media literacy sessions, and eight sessions during which the students developed a media campaign for their parents (who were then exposed to the campaign). Intervention group also invited to two family fun nights at their school.  Control group did not receive after-school program but were invited to two family-fun nights at their school. |
| **Outcomes** | FV availability at the home: the index measuring FV availability consists of nine items and response options were "yes" or "no". Item responses were summed and the score was used for analyses. A lower score on this index was indicative of greater availability.  Post-intervention comparisons between intervention and control parents - significant differences between groups  Parent Scales: Mean +/- SD  **Availability**  Pretest: Intervention (n = 13): 11.54 +/- 1.61; Control (n = 15): 10.87+/-0.83; t value: -2.55; df: 18; p (two-tailed): 0.02  Post-test: Intervention (n = 13): 9.78 +/-0.67; Control (n = 15): 10.64+/- 0.81  **Children's consumption**  Pretest: Intervention (n = 13): 1.94 +/-1.01; Control (n = 15): 2.06+/-1.83; ANCOVA F test: 0.484; df: 15; p: 0.493  Post-test: Intervention (n = 13): 1.68 +/1.13; Control (n = 15): 2.00+/- 1.10  **Children's self-efficacy**  Pretest: Intervention (n = 13): 140/81 +/- 24.87; Control (n = 15): 115.65+/-34.65; ANCOVA F test: -0.260; df: 26; p: 0.619  Post-test: Intervention (n = 13): 130.64+/-31.69; Control (n = 15): 119.25+/- 34.29  **Children's motivation (lower score indicates greater motivation)**  Pretest: Intervention (n = 13): 12.00 +/- 3.62; Control (n = 15): 2.06+/-1.83; ANCOVA F test: 7.370; df: 21; p: 0.013  Post-test: Intervention (n = 13): 12.9+/-5.83; Control (n = 15): 14.41+/- 5.45 |

**Thibault 2010**

| **Methods** | Before after, but used different schools and teachers |
| --- | --- |
| **Participants** | 455 were invited schools; 343 participated; 257 schools reported data first time around. Intervention was delivered through the school administration. Teachers were target audience. |
| **Interventions** | Daily intervention, not clear what they did but they were trying to change teachers giving snacks to children or change snack composition. Duration not described. |
| **Outcomes** | Questionnaires to teachers: Improvement of snack composition (more fruit and milk products) Comparison of proportion of teachers between first (2004-2005) and second survey (2007-2008)  Teachers attitudes towards need for morning snack  Pretest - 22% reported not needed. Posttest- 44% reported not needed; p<0.001 |

**Thompson 2009**

| **Methods** | Cluster-controlled |
| --- | --- |
| **Participants** | Forty-two Boy Scout troops (n = 473, 10-14 year-old Scouts) in Houston, TX |
| **Interventions** | Treatment group participated in an intervention to increase FV consumption (5 A-Day), control group participated in mirror-image intervention to increase physical activity with same general structure (55 minutes of weekly programming - 30 minutes in troop; 25 minutes online)  Treatment group: In-troop activities taught functional knowledge, skills to enhance self-efficacy and troops participated outcome-expectancy-related comments to enhance motivation to consume FV, simple recipes and recipe tastings; online activities encouraged Scouts to log-on to study website at last twice a week to participate in behaviour change program, set goals and report on goals |
| **Outcomes** | Results from fruit/juice consumption and psychosocial measures stratified by intervention group (5 A Day, control)  Availability of fruit/juice (items): Means (standard errors)  **5 A Day**  Baseline (n = 224): 9.0 (0.4)  Immediately post (n = 235): 10.9 (0.4)  6 months post (n = 213): 10.0 (0.4)  **Control**  Baseline (n = 228): 9.1 (0.3)  Immediate post (n = 234): 9.7 (0.3)  6 months post (n = 219): 10.0 (0.3)  F= 4.76, p=0.009 Significant change from baseline to immediate post test.  Availability of low-fat vegetable (items): Means (standard errors)  **5 A Day**  Baseline (n = 224): 8.3 (0.3)  Immediately post (n = 230): 9.4 (0.3)  6 months post (n = 213): 8.9 (0.3)  **Control**  Baseline (n = 228): 8.2 (0.3)  Immediately post (n = 234): 8.7 (0.3)  6 months post (n = 219): 9.0 (0.3)  The 5AD group reported a mean increase of 1.87 (0.3) available FV items, as compared to a mean increase of 0.58 (0.3) items in the control group.  Consumption of fruit/juice (servings): means (standard error)  **5 A Day**  Baseline (n = 224): 2.5 (0.1)  Immediately post (n = 235): 3.5 (0.1)  6 months post (n = 213): 2.8 (0.1)  **Control**  Baseline (n = 228): 2.3 (0.1)  Immediate post (n = 234): 2.9 (0.1)  6 months post (n = 219): 3.0 (0.1)  F= 5.90, p=0.003 Statistically significant change from baseline to immediate post.  Self-efficacy for fruit/juice intake: Means (standard errors)  **5 A Day**  Baseline (n = 224): 27.1 (0.5)  Immediately post (n = 235): 28.4 (0.5)  6 months post (n = 213): 28.0 (0.5)  **Control**  Baseline (n = 228): 27.2 (0.4)  Immediate post (n = 234): 27.0 (0.4)  6 months post (n = 219): 27.0 (0.5)  F= 2.70, p=0.068 Statistically significant change from baseline to immediate post. |

**Walsh 2003**

| **Methods** | Quasi-experimental |
| --- | --- |
| **Participants** | 6 communities  **4 intervention:**  Heidedal: n1=208; n2=208  Ritchie: n1=122; n2=124  Jagersfontein: n1=116; n2=96  Fauresmith: n1=85; n2=85  2 control:  Trompsburg: n1=62; n2=81  Bethulie: n1=69; n2=78 |
| **Interventions** | Intervention group received a food aid program (National Nutrition and Social Development Programme) as well as Nutrition Education Intervention Programme (NEIP) Programme. NEIP used nutrition advisors (local persons appointed in communities where infant morbidity and mortality rates were high) as community health workers with nutrition as their first priority; they were trained to improve the community's knowledge of balanced, economical nutrition using the three food group approach and were involved in service functions such as nutrition monitoring and establishing self-help programmes, such as communal gardens, play groups for children (concentrating on nutrition-related activities) and women's sewing groups.  Control group received only the food aid program. |
| **Outcomes** | Grow your own vegetables? (Yes)  **Intervention**  Heidedal: % initial: 12.0; % second: 23.6; Difference in %: 10.6; 95% CI 3.2-18.0  Ritchie: % initial: 11.5; % second: 55.6; Difference in % 44.1; 95% CI 33.7-54.1  Jagersfontein: % initial: 3.4; not reported because of unreliability  Fauresmith: % initial: 10.6; % second: 60.0; Difference in %; 29.4; 95% CI 371.-61.7  **Control**  Trompsburg: % initial: 14.5; % second 25.9; Difference in %: 11.4; 95% CI -1.6-24.4  Bethulie: % initial: 31.3; not reported because of unreliability  Knowledge  Multiple single questions were scored as to the percent who answered correctly in each community before and after. No overall score was calculated or mean difference. |
